# Youth hedonistic behaviour: moderating role of peer attachment on the effect of religiosity and worldview

**DOI:** 10.1080/02673843.2014.942793

**Published:** 2014-08-22

**Authors:** Siti Raba'ah Hamzah, Turiman Suandi, Steven Eric Krauss, Azimi Hamzah, Ezhar Tamam

**Affiliations:** ^a^ Department of Professional Development and Continuing Education, Faculty of Educational Studies, Universiti Putra Malaysia, Selangor43400, Malaysia; ^b^ Institute for Social Science Studies, Putra Infoport, Universiti Putra Malaysia, Selangor 43400, Malaysia; ^c^ Department of Communication, Faculty of Modern Languages and Communication, Universiti Putra Malaysia, Selangor 43400, Malaysia

**Keywords:** peer attachment, hedonistic behaviour, youth, religiosity, worldview

## Abstract

This study was carried out on the moderating effect of peer attachment on the relationships between religiosity and worldview, and on how hedonistic behaviour among Malaysian undergraduate students is shaped by such influences. With regard to peer attachment, the study focused on the influences of communication, trust and alienation among youth. Bronfenbrenner's theory of human ecology and Armsden and Greenberg's attachment model were used as the framework. Drawing on a quantitative survey of 394 Malaysian university students (*M* age = 21.0, SD = 0.40), structural equation modelling and path analysis revealed a significant relationship between worldview and hedonistic behaviour. Peer attachment moderated the relationships between religiosity and religious worldview. The results further showed that the unique moderating effect of the lower level of attachment with peers is positively related to the hedonistic behaviour. Implications from the findings are discussed.

## Introduction

Youth as a phase of life has been defined differently across time and according to different cultures. In Malaysia, youth falls within the age group between 15 and 40 years. This group of more than 11.6 million, or 43.6% of the total population, outnumbers children or adults in Malaysia (Department of Statistics, 2010). With Malaysia undergoing a process of rapid development and modernisation, the changes to the socio-demographic and cultural environment alter the social process, especially for youth. This has resulted in significant changes in social development for youth from different backgrounds, making it necessary for them to adapt to a rapidly changing development environment.

The behaviour of young people is reflexively influenced by their surrounding environment. At the same time, youth are active agents in forming their own environment (Bronfenbrenner, 1989). A study on Malaysian culture and youth subcultures (Azimi, Turiman, & Ezhar, 2000) indicated the emergence of new values and cultural patterns among young people. These include individualism and materialism, religious orientation, the orientation to foreign cultures, identity changes, communication behaviour and media habits, leisure activity, health habits and career aspirations.

In Malaysian universities, students' capability to adjust to a new lifestyle plays an important role in determining their academic success. Research has revealed that the ability to adjust within campus settings is associated with certain psychosocial factors including support from parents and peer attachment (Maria, Elias, Mahyudin, & Uli, 2009). A majority of Malaysian university students stay with their peers throughout the year while attending institutions of higher learning. The Ministry of Malaysia Higher Education (2010) reported that only 10–20% of 1.2 million university students in Malaysia live with their parents. Although parents generally have a powerful influence on their children's behavioural development, peers have been shown to exert an important impact as well.

Based on the second level of Bronfenbrenner's (1979) ecological model, much focus has been directed to relationships with peers as socialising agents influencing the development of youth behaviour. However, much of the research on the role of peers in the development of youth behaviour is limited to Western samples. Hence, for the purpose of this study, a Malaysian setting was selected to determine the moderating effect of peer attachment on the hedonistic behaviour of Malaysian undergraduate students.

In the context of youth behavioural development, there are many different explanations or theories for why some youth become delinquent or experience psychological disorders while others succeed in spite of adverse circumstances. Bronfenbrenner's ecological model of human development was selected as the focus for this study. This theory is based on the empirical studies of researchers from diverse disciplines who had studied the background of children's environment and their development for many years. Bronfenbrenner argues that in order to understand human development, we must consider the entire ecological system which consists of five socially organised subsystems that help, support and guide human growth. The basic understanding of this theory is the relationship between the development of an individual and the impact of his/her environment as influenced by family, school and peers, all of which contribute to new culture, economy, custom and bodies of knowledge.

In Malaysia, as with many other countries undergoing the process of modernisation and development, reforming contemporary lifestyles of youth morphs fluidly with the changing social environment. Youth behaviour development could be impacted even more by a multicultural environment. In Malaysia, apart from the dominant Malay, Chinese and Indian populations, there are many others races such as Iban, Kadazan, Melanau and other ethnic groups. Within these groups, there is diversity in culture, custom, language, social class and caste, education, religion and ethnicity. These differences are valuable aids to the broadening of outlook and behaviour development (Keats, 2000). Research on Malaysia Youth Index by the Malaysian Institute for Youth Research in Development (2011) shows that there are nine indicators of the overall health of youth and their well-being, namely self-development, social relationship, identity, self-potential, leisure time, health, media penetration, deviant behaviour and economic well-being. These indicators not only help to monitor current youth lifestyle and thinking patterns but also help stakeholders identify areas and strategies to improve the quality of life and well-being of Malaysian youth.

Research by Cheng (2013) summarises youth generational perspectives on life across Malaysia – that they are comfortable with life under parental protection and security, they have an unclear vision towards life, they follow modern and fashionable trends, they like social networking and are highly flexible to change and adapt to new cultures and surroundings. Practising hedonistic lifestyles by youth is not a new phenomenon in Malaysia. The empirical association between the level of hedonistic behaviour and the demographic factors among undergraduate students in Malaysia was investigated in a study by Hamzah, Krauss, Suandi, Hamzah, and Tamam (2013). The result showed that the majority of youth at medium and high levels of hedonistic behaviour are based on the criteria suggested by Zimbardo and Boyd (1999).

There are various connotations of the term ‘hedonistic behaviour’ (Chaudhuri, Aboulnasr, & Ligas, 2010; Jantzen, Fitchett, Østergaard, & Vetner, 2012; Veenhoven, 2003). Some researchers are of the opinion that hedonistic behaviour is what is shaping the contemporary lifestyle of young people (Andersson, 2011; Itafarida, 2008; Thorpe, 2012; Veenhoven, 2003). In this study, two definitions of hedonistic behaviour are examined. First, from an ethical perspective, it is a prescriptive theory which states that as a philosophy, the seeking of pleasure is the highest good and moral values are derived from within the individual (Crisp, 2006; Sobel, 2002). The second definition is that psychologically, as a descriptive theory, hedonism views the pursuit of the thrill of driving to attend to one's self-devotion as a way of life (Bentham, 1789 in Weijers, 2011; Feldman, 2008).

According to Hofstede's Taxonomy of Culture, the hedonistic behaviour could be reclaimed under the sixth dimension recommended by Minkov (2007) labelled as indulgence versus restraint. Indulgence allows relatively free gratification of basic and natural human desires related to enjoying life and having fun. Restraint controls gratification of needs and regulates it by means of strict social norms. Research by Minkov (2007) on indulgence versus restraint for 93 countries showed different results among the societies. Indulgence tends to be dominant in South and North America, western Europe and in parts of Sub-Sahara Africa. Restraint on the other hand prevails in eastern Europe, in Asia and in the Muslim world. This research suggests that the difference between indulgent and restrained societies is based on several criteria such as ‘higher percentage of people declaring themselves very happy’ versus ‘fewer very happy people’, ‘higher importance of leisure’ versus ‘lower importance of leisure’, ‘in wealthy countries, lenient sexual norms’ versus ‘in wealthy countries, stricter sexual norms’ and ‘perception of personal life control’ versus ‘a perception of helplessness’. These findings provide initial support for the assumption that a Muslim country such as Malaysia is featured with the restraint dimension.

Moreover, in this study, hedonistic behaviour is characterised by openness to pleasurable experiences and tending to indulge one's own desires. Zimbardo and Boyd (1999) argue that hedonistic behaviour reflects attitudes towards time and life, whereas Veenhoven (2003) is of the opinion that hedonistic behaviour applies in particular to the pursuit of sensory pleasures such as drinking alcohol, abundant sex and undertaking risky activities to maximise happiness. As such, the social concern is that hedonistic behaviour expressed as the pursuit of luxurious life styles erodes social bonds when pleasure-seeking behaviour makes people less sensitive to the needs of others, and might lead to moral decay.

Hence, this study also addresses the methodological concern of the types of measures used to assess the influence of peer attachment (with regard to communication, trust and alienation) on the relationship between religiosity and worldview in assessing hedonistic behaviour among Malaysian undergraduate students.

## Religiosity and youth behaviour

Religiosity or religious commitments is defined as ‘the extent to which an individual is committed to the religion he or she professes and its teachings, such as the individual's attitudes and behaviours reflect this commitment’ (Johnson, Jang, Larson, & De Li, 2001, p. 25). Religiosity and behaviour development are particularly important issues for emerging adults because these individuals are in the process of exploring new worldviews (Arnett, 2000). A number of studies have examined the associations between religiosity and youth behaviour, religiosity and deviance (Albrecht, Chadwick, & Alcorn, 1977), religion's role in promoting health and reducing risk among youth (Wallace & Forman, 1998), social context in the development of adolescent religiosity (Regnerus, Smith, & Smith, 2004), religion/spirituality and adolescent health outcomes (Cotton, Zebracki, Rosenthal, Tsevat, & Drotar, 2006), religion as a resource for positive youth development: religion, social capital and moral outcomes (King & Furrow, 2008), religiosity of adolescents, their friends and network associates (French, Purwono, & Rodkin, 2012), and adolescent religiosity and psychosocial functioning (Stolz, Olsen, Henke, & Barber, 2013). Most of these studies reveal that religiosity has a powerful influence on youth behaviour. It has become widely accepted that youth is a significant period for the development of religiosity (Desmond, Morgan, & Kikuchi, 2010).

Studies on the relationship between behaviour and religiosity were empirically initiated in the West. Thus, it is not surprising that the literature on the relationship between religiosity and the worldview of young people in Malaysia is scarce. Nevertheless, studies have shown that religiosity is significantly correlated with behaviour; a stronger and more positive affectional bond with parents and peers is linked to and may facilitate socialisation-based religiosity (Krauss et al., 2013). Religion provides youth with a set of beliefs and values that prohibit behaviours considered immoral, illegal or antisocial (Wallace & Williams, 1997).

The relationship between religiousness and the development of behaviour has received growing attention by researchers. Several studies have linked religious attachment with positive behaviour development, including religiousness as a buffer against risk behaviour and support for positive attitudes and actions among youth. Pargament and Park (1995) suggest that religious involvement acts as a source of support, encouragement, coping and resilience. Youth who perceive religion as important are active in religious worship and activities, showing that the perceived importance of religion and participation in religious activities are associated with decreased risk behaviours. Looking at 10 risk behaviours, religiosity variables are consistently associated with reduced risk behaviours such as smoking, alcohol use, truancy, sexual activity, marijuana use and depression (Sinha, Cnaan, & Gelles, 2007).

Local studies show that cultural and religious norms, values and expectations among Malays are influenced by Islam, which also has an effect on the respondents' moral judgement (Jaafar, 2004). Krauss et al. (2006) argue that Malay youth in Malaysia struggle to blend tradition and modernity with proponents of strict secularisation and Islamic factions within the country. The Malaysian government has adopted a moderate form of Islam to balance between the traditional and modern manifestations of Islam.

## Worldview development and youth behaviour

Worldview, as related to the sciences, ethics, arts, politics and religions, is an integral part of all cultures (Aerts et al., 2007). Strongly motivating, it inspires future directions with a socially shared view towards a sense of direction, confidence and self-esteem. Worldview combines values, attitudes, perceptions, assumptions and ideas to form a perspective of life for the individual. It also incorporates formulations and interpretations of past, present and future with a complex conceptual framework, depending on the beliefs of the individual. The worldview concept is based on Bowlby's (1969) attachment theory which postulates the human inclination to make strong affectional bonds with the environment, and explains the many forms of emotional and personality development. However, worldview has a broader construct that is influenced by more interactions around the world. Hence the thinking patterns and behaviour with regard to religious worldview are factors which are investigated in this study.

Religious worldview is believed to have an influence on the religiosity formation of youth. According to Deckard and Dewitt (2003), there are three important factors which influence youth worldview, namely theology, science and age. Theological aspects encourage the formation of a belief system to appreciate nature and participate in the cycles of the ecological model (Bronfenbrenner, 1989). According to Kearney (1984, p. 1), worldview is a ‘culturally organized macro thought: those dynamically inter-related basic assumptions of a people that determine much of their behavior and decision making, as well as organizing much of their body of symbolic creations … and ethnophilosophy in general.’ The formation of a worldview is associated with cognition, learning, perception and behaviour, and environment. The driving force behind the development of a worldview is the need to relate to the outside world. As aptly stated by Ross (1962), man's ‘experience is useless unless interpreted’. Therefore, beginning from childhood, each person interacts with his physical and social environment, and through this myriad of environmental interactions, worldview presuppositions are unconsciously constructed. The process occurs over a long period of time, with the formative, young adult years being of utmost importance. Through years of schooling, formal education contributes to worldview development, which in turn, provides a foundation upon which cognitive frameworks are built during the learning process. From this, it is assumed that the worldview is the system, which is always subjected to changes, of implicit and explicit views and feelings of an individual in relation to human life.

This paper also examines the moderating effect of peer attachment on the relationship between religious worldview and youth hedonistic behaviour.

## Peer attachment and its influence on youth behaviour

Attachment would be defined as perceived relational bond existing between two individuals. According to Armsden and Greenberg (1987), attachment involves an emotional bond that is experienced with a substantial degree of intensity. In this study, peer attachment is the perception of a relational bond experienced between individuals of equal relational standing and established through proximity and time investment. Nevertheless, peer attachment is expressed through varying levels of trust, communication and alienation (Armsden & Greenberg, 1987).

An important theoretical line of inquiry related to behavioural development and formation is that of the peer attachment theory. According to Huang, Wang, and Shi (2012), attachment and relationship with peers among youth have proven to be significant in influencing behaviour. A peer is considered the family's substitute outside the home, and is one of the transition mechanisms from childhood into teenage years and adulthood. Friends provide support socially and academically for sharing experiences, acting as socialising agents and as a platform to expand social circles. Peer influence also shapes youth personality and individuality (Jas Laile Suzana, 2008). Many studies show that teenagers with confidence and trust in their relationships with their peers tend to have strong resolution, good self-control and are able to manage themselves (Armsden & Greenberg, 1987).

Research also shows that close peer relationships have positive psychological influences as well as increasing happiness (Durlak, Weissberg, & Pachan, 2010; Piaget, 1965). Inadvertently, this also contributes to the development of important values which generate happiness and self-confidence, and which in turn motivates youth to display positive attitudes and behaviour. According to Armsden and Greenberg (1987), attachment with peers happens in three dimensions: trust, communication and alienation.

Peers often have considerable influence on youth behavioural development. Kandel (1978) reports that youth attitude and behaviour mirror each other. A number of studies reveal the important roles peers play as agents of socialisation and expanding social connections. Dykas, Ziv, and Cassidy (2008) revealed that attachment was linked to a moral transgression (i.e. aggression against peers), and this finding provides new insight into how attachment processes are linked to adolescent moral development. Findings indicate that peers elicit more negative and deviant behaviours than positive ones (Albert & Steinberg, 2011; Brechwald & Prinstein, 2011; Zahrt & Lange, 2011). Similarly, Tomé et al. (2012) find that peers have a direct effect in encouraging violence and negative behaviours. Many studies also indicate that youth with friends involved in risky behaviours are more likely to be engaged in similar risky activities (Glaser, Shelton, & Bree, 2010). Other reports indicate that students with lower quality attachment relationships are more likely to bully others and be the victims of bullying than their peers with higher quality attachment relationships (Walden & Beran, 2010).

## Peer attachment as a moderating factor

Theory and research have suggested that attachment to peers can moderate youth behaviour. Past studies indicate that levels of emotional well-being, beliefs about self, and values for prosocial forms of behaviour and social interaction are stronger in youth with positive peer attachment (Rubin, Bukowski, & Parker, 2006). Liu (2011) found negative relationships between peers with youth involved in delinquency. The study also found that youth with depression and a negative outlook on life often had weak attachment with peers. Earlier research by Urberg, Luo, Pilgrim, and Degirmencioglu (2003) also indicates that attachment with peers is a moderating factor in determining youth involvement with delinquent behaviours and activities. Studies in Malaysia have shown significant relationships between peer attachment and hedonistic behaviour which contribute to pleasure seeking and lead to moral degeneration (Hamzah et al., 2013).

Research illustrates that the more involved youth are with negative activities, the less likely they will have positive attachment with parents and peers. Lynam, Loeber, and Stouthamer-Loeber (2008) investigated the relationship between adolescents and parents, using peer attachment as the moderator in affecting the development and shaping of youth behaviour in relation to juvenile activities. Research continues to demonstrate the negative implications arising from negative relationships between peers and youth, which lead to antisocial behaviours and a rise in social problems among youth (Albert & Steinberg, 2011). Another important research exposed that the close relationship serves as a buffer against delinquency for individuals with non-dismissing of attachment especially with peers (Mcelhaney, Immele, Smith, & Allen, 2006).

## The current study

Religiosity and worldview, reinforced by attachment with peers, play an important role in shaping youth behaviour. However, as much of the research on peer attachment and youth behaviour is carried out in the West and the USA, it is not possible to make use of national data-sets, which do not exist in Malaysia. Furthermore, little effort has been made by researchers to delve into youth hedonistic behaviour in the context of a developing country. Hence, using the theory of human ecology (Bronfenbrenner, 1989) as the framework, this study aimed to determine the extent of the influence of religiosity and worldview on hedonistic behaviour, with particular focus on the moderating effect of peer attachment (Armsden & Greenberg, 1987).

The focus of this study was on peer attachment among undergraduate students in relation to hedonistic behaviour. It was hypothesised that peer attachment (in the areas of communication, trust and alienation) would moderate the relationships between religiosity and worldview on the hedonistic behaviour of Malaysian undergraduate students. Accordingly, the following hypotheses were tested:

Hypothesis 1: There is a significant relationship between worldview and hedonistic behaviour.
Hypothesis 2: There is a significant relationship between religiosity and hedonistic behaviour.
Hypothesis 3: There is a significant relationship between religiosity and worldview towards peer attachment had an indirect effect on hedonistic behaviour.

To broaden the scope of peer attachment measures, this study included three peer attachment dimensions, namely trust, alienation and communication, which drew on the peer attachment theory (Armsden & Greenberg, 1987). The study also took into account findings on universal religiosity personality (Krauss, Azimi, & Fazilah, 2007), worldview which looks at theology and science (Deckard & Dewitt, 2003) and hedonistic behaviour (Zimbardo & Boyd, 1999).

## Method

### Participants and procedures

Selected undergraduate students of higher education institutions in Malaysia comprised the sample for this study. The stratified random sampling technique was used to ensure the representativeness of the chosen sample. A total of 394 respondents from public and private universities were selected. The sample consisted of 182 (46.1%) males and 212 (53.9%) females, with the majority, i.e. 305 (77.5%), living in town/suburban areas and 89 (22.5%) from urban areas. The mean age of the participants was 21 years (SD = 0.40), with a range of 18–22 years. Data were collected using the survey method. After permission was granted by the lecturers of each university, the questionnaires were given to the respondents and collected after 30–45 minutes. The data were analysed using SPSS v. 20. Descriptive statistics were used to compare mean scores for the study variables. Structural equation modelling (SEM) and path analysis were employed to test the hypothesised relationships.

#### Measures

The study utilised a questionnaire that was divided into five parts: demographics, worldview and religiosity (Krauss et al., 2007), peer attachment (Armsden & Greenberg, 1987) and hedonistic behaviour (Zimbardo & Boyd, 1999). All variables were tapped with self-report questionnaires. A five-point Likert-scale format was used, from 1 (strongly disagree) to 5 (strongly agree).

The Universal Religiosity Personality Inventory (Krauss et al., 2007) measures religiosity in the context of prosocial and ritual behaviours. Sample items on the prosocial behaviour scale included ‘I immediately apologise if I wrong someone’, ‘I try to smile as much as possible’ and ‘I speak politely to my parents’. For the ritual behaviour scale, sample items included ‘I make effort to deepen my understanding of law/rules/teaching/precepts of my religion’, ‘I invite others to obligatory prayer’ and ‘I perform my work duties enthusiastically because of my religion’. Although many studies conducted in the West have included prosocial and ritual behaviours, little attempt has been made to measure religiosity in the context of a multiracial community such as in Malaysia where there are unique multicultural ethnic representations, with Muslims as the majority (49%), Buddhists (32%), Hindus (8%) and others (11%), which include Christians and Sikhs.

The section on peer attachment in the questionnaire for this study contained items adapted from the Inventory of Parent and Peer Attachment by Armsden and Greenberg (1987), a 25-item self-report aimed at evaluating the quality of adolescent attachment to peers. Each of the instruments consists of Likert-type statements that assess how adolescents evaluate their relationship with their peers on the following subscales: mutual trust, quality of communication and the extent of felt alienation. In the section to evaluate peer relationship, examples of items for the communication subscale were the following: ‘I like to get my friends’ point of view on things I am concerned about' and ‘When we discuss things, my friends care about my point of view.’ Further examples of items for trust subscale are the following: ‘My friends understand me’ and ‘My friends accept me as I am.’ In addition, examples of items for alienation subscale are the following: ‘My friends don't understand what I am going through these days’ and ‘I feel angry with my friends.’

The worldview measures in this study were adapted from the instrument by Deckard and Dewitt (2003). Sample items of theology scale included: ‘I believe there is only one God is the source of all creation’, I believe that man is the best creation of God', I believe that God knows all the developments taking place in this world', ‘I believe that my future has been determined’, ‘I believe people need to change for the better’, ‘I believe there is good in every weakness’, ‘I believe in time, space and opportunity always exist in life’, ‘I believe I can identify the good and the bad things in my life’, ‘I believe life has a specific purpose’ and ‘I always think about the moment of my death.’

With regard to the section on hedonistic behaviour, the questions were adapted from Zimbardo Time Perspective Inventory (Zimbardo & Boyd, 1999), a fundamental dimension in the construction of present hedonistic behaviour characterised by an orientation towards present enjoyment, pleasure and excitement. It reflects a hedonic risk-taking attitude towards time and life, and includes such diverse items as ‘taking risks keeps my life from becoming boring’, ‘I do things impulsively’, ‘I often follow my heart more than my head’ and ‘when listening to my favourite music, I often lose all track of time’ and ‘It is important to put excitement in my life.’

## Results and discussion

Many findings show that the combination of Time Perspective Inventory and Religiosity Personality Inventory remains a uniquely independent contribution to the many factors which relate to youth behavioural development (Bosnia & Kunnen, 2001; Kroger, 2000). In order to ascertain whether there was configuration of relationships proposed in the conceptual model for religiosity, worldview and peer attachment, a multiple group analysis in SEM was performed. Results of the SEM test of the model between religiosity and worldview towards hedonistic behaviour showed that the model fitted the data. The following general guidelines for fit indices were used: goodness-of-fit index (GFI) statistic, root mean square of error approximation (RMSEA) for absolute fit measure, normed fit index (NFI), Tucker–Lewis index (TLI) and comparative fit index (CFI) for incremental fit measure, and normed chi square (χ^2^/df) for parsimonious fit measure (Browne & Cudeck, 1993; Byrne, 2010; Hair, Black, Babin, & Anderson, 2010; Hu & Bentler, 1999).

As shown in Table 1, the results indicated a good fit of the model; the ratio of χ^2^ to degrees of freedom and CFI are all in acceptable range (χ^2^ = 685.521, *p* = 0.000, χ^2^/df = 2.034, GFI = 0.930, CFI = 0.944, incremental fit index (IFI) = 0.944, TLI = 0.937, RMSEA = 0.050). The RMSEA's 90% confidence interval was from 0.05 to 0.08. GFI, CFI, IFI and TLI were more than 0.90, indicating a good fit for all variables.

**Table 1:** Test of the model fit indices for all variables.

GOF index	χ^2^/df/CMIN	GFI	CFI	IFI	TLI	RMSEA
Value	2.034	0.930	0.944	0.944	0.937	0.050

Note: GOF, goodness of fit; CMIN, chi-square minimum.

**Figure 1:**
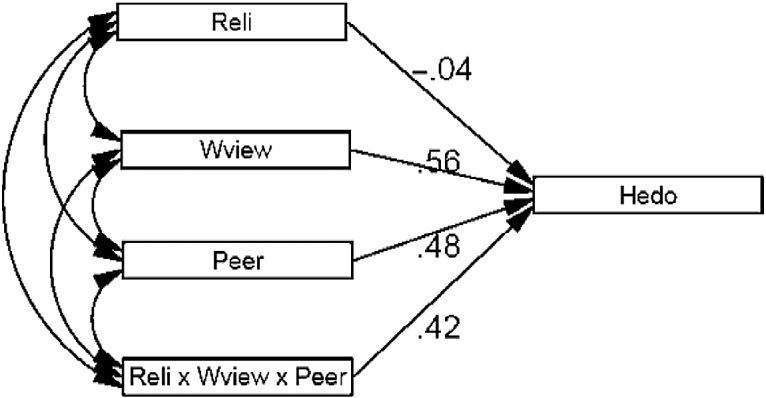
A model showing the influences of worldview, religiosity and peer attachment on hedonistic behaviour. Note: reli, religiosity; wview, worldview; peer, peer attachment; hedo, hedonistic behaviour.

On examining the hypotheses, H1 demonstrated a significant relationship between worldview and hedonistic behaviour. Hypothesis 1 was, therefore, supported. The path analysis between religiosity and hedonistic behaviour also demonstrates a significant relationship and Hypothesis 2 was, therefore, supported. Concerning the moderating effects of peer attachment on the development of hedonistic behaviour based on feedback regarding communication, trust and alienation, an overall structural model was developed and tested. The hypothesised model in this study was grounded on the literature outlined in the previous section. The conceptual model took into account the influences of religiosity and worldview on the shaping of hedonistic behaviour, with a moderating effect by peer attachment. The path diagram delineating the conceptual model is shown in Figure 1.

A mean-split approach was used to break the study sample into two groups according to their mean scores on the peer attachment measure. The data above the mean were defined as high peer attachment, and the data below the mean as low peer attachment. A two-group analysis of moment structures model was used subsequently so that it could be determined whether or not there was any significant difference in structural parameters between the high peer attachment group and the low peer attachment group. Differences in the χ^2^ values between the two models determined whether the peer attachment had a moderating effect on the relationship between worldview and religiosity on hedonistic behaviour.

A direct comparison of the fit indices was conducted for the two opposing models. We hypothesised that the relationship between religiosity and worldview towards peer attachment had an indirect effect on hedonistic behaviour in Hypothesis 3. Table 2 outlines the χ^2^ GFI statistics and baseline comparison fit indices. The baseline comparison fit indices of IFI, TLI and CFI for both models were above 0.90 even though the χ^2^ tests for the models recorded poor fit. The RMSEA values for group variant and group invariant path models were 0.044 and 0.051, respectively, suggestive of adequate fit. The Akaike's Information Criterion (AIC), which has a bearing on model parsimony and model fit, was then used to compare the model fit for the two models. A simple and well-fitted model is indicated by low scores. The AIC value for the group variant model (847.591) was lower than that of the group invariant model (941.388), implying that the group variant model outweighed the other one with a more parsimonious model fit. As a result, the two models differed significantly in their goodness-of-fit. Hence, peer attachment moderated the causal relationships in the model because a more parsimonious and better fitting was found in the group variant model for the group of higher attachment level than that for the group of lower attachment level to peers. The findings showed that peer attachment moderated the relationships between religiosity and worldview on hedonistic behaviour.

**Table 2:** Results of moderation test of peer attachment on hedonistic behaviour.

Model	χ^2^	df	*p*	CMIN/df	RMSEA	AIC	χ^2^	Test of moderating
Constrained	845.388	414	0.000	2.042	0.051	941.388		
Unconstrained	655.591	366	0.000	1.791	0.044	847.591	189.797	Significant and moderate effects

Results of the multi-group testing for moderation effects are shown in Table 3. The hypothesis testing the moderating effect of peer attachment on the relationship between religiosity and worldview and the development of hedonistic behaviour was supported. The group with high level attachment indicated a mean score of 4.00 (SD = 0.30), while mean score for the group with low level attachment was 2.66 (SD = 0.32). We employed χ^2^ difference tests to compare the two groups. The results show that the relationship between religiosity and hedonistic behaviour is not moderated by the higher level of peer attachment, but it was moderated by the lower level of attachment with peers. Thus, examining the path coefficient values between higher level of attachment with peer (β = –0.025, *t* = –0.171) and lower level of attachment with peer (β = 0.636, *t* = 5.353) shows some differences in the various paths. The second path showed that the relationship between worldview and hedonistic behaviour is moderated by the lower level of attachment with peers (β = 0.349, *t* = 4.441) and not moderated by the higher level of attachment (β = 0.049, *t* = 0.327). The results indicate that for those with a lower level of attachment with peers, the relationship between religiosity and worldview with hedonistic behaviour is positive, while for those with high attachment, the relationship is negative. These results imply that for adolescents with low attachment to peers, religiosity and worldview do not buffer against engagement in hedonistic behaviour, while for those with high attachment, there is a buffering effect between religiosity/religious worldview and hedonistic behaviour.

**Table 3:** Comparisons of path coefficient and *t*-value (critical ratio, CR) for high and low level of attachment.

	Higher level of attachment			Lower level of attachment		
Paths	Estimates (β)	*t*-Value CR	*p*	Estimates (β)	*t*-Value CR	*p*
Religiosity → hedonistic behaviour	− 0.025	− 0.171	0.864	0.636	5.353	0.000
Worldview → hedonistic behaviour	0.049	0.327	0.744	0.349	4.441	0.000

The study results provide support for peer attachment as a moderator indicating that higher attachment with peers is an important positive contributor to adolescent behaviour by acting as a buffer against engagement in hedonistic behaviour. The results support previous studies showing that stronger peer attachment results in stronger positive relationships (Cullman, 2006). Stronger attachment to peers provides a source of emotional support, companionship, personal validation and helping in solving problems, especially during adolescence, a basis for identity development. The results also confirm past research by Salzinger, Feldman, Rosario, and Ng-Mak (2010) that attachment with peers is a moderating factor in developing positive youth attitudes.

For the other group, however, low attachment appears to contribute to hedonism, perhaps due to the lack of positive social anchoring that comes with high quality peer relationship. When peer relationships are not grounded in trust and warmth, young people will seek pleasure-based activities to compensate for the lack of fulfilment. In terms of religious attitudes and behaviour, the better the quality of the relationships the more inclined the individual is to accept and adopt the religious perspectives of their peers. Furthermore, according to Waldrip, Malcolm, and Jensen-Campbell (2008), not having good relationships with friends on whom they can rely can have negative mental health outcomes, including emotional and behavioural adjustment difficulties which can influence their involvement in hedonistic activities. The quality of friendships and the types of activities involved in with those friends can direct young people towards negative behavioural outcomes (Hartup, 1996).

This study provides evidence for the independent links between religiosity, worldview and hedonistic behaviour, while also providing support for peer attachment as a moderator. These findings raise two possibilities. First, a number of researchers have argued that peer attachment may be salient in its influence on antisocial behaviour, as mentioned by Albert and Steinberg (2011). Even though religiosity and religious worldview can act as a source of support for positive attitudes (Pargament & Park, 1995), neither variable contributed to the development of positive behaviour among those with low peer attachment in our sample. On the other hand, high levels of peer attachment influenced the formation of positive behaviour development resulting from religiosity and religious worldview. Therefore, peer attachment might provide a certain socio-emotional competence, such as prosocial behaviour and close peer relationships have positive psychological influences as well as increasing happiness (Durlak et al., 2010; Scholte & Van Aken, 2006).

## Conclusion

In this study, researchers developed and tested a model in which religiosity, worldview and peer attachment were hypothesised to affect hedonistic behaviour, both directly and indirectly. The findings revealed that a person's worldview will have an influence on the development of his or her hedonistic behaviour. It can also be concluded that a person's religiosity also has an influence on the development of his or her hedonistic behaviour. Furthermore, peer attachment plays a significant role in moderating the overall relationship between worldview and religiosity, and the hedonistic behaviour of a young person. This implies that peers have a significant influence on a young person's involvement in hedonistic behaviour.

Although peer attachment is indirectly assessed in this study, the inclusion of this factor as a separate indicator lends support to the Bronfenbrenner's ecological model of human development (1979). According to Armsden and Greenberg (1987), a positive relationship between peers improves psychological well-being in adolescents. The findings of this study can provide a basis for parents and other actors in young people's social ecologies, as suggested by Bronfenbrenner (1989), to recognise peers as an important factor contributing to the formation of youth behaviour and personality development. The authors recommend future research that incorporates a combination of quantitative and qualitative methods to obtain more contextual information about the nature of Malaysian youth peer attachment.

Future research on the role of peer attachments would be served through longitudinal data to shed further light on the development process, leading to the formation of the youth behaviour across the lifespan. Additional measures on peer attachment and religiosity, along with additional ecological model of human development system measures that include the roles of family are important considerations for the Malaysian societies, where the family role plays a major agent for the socialisation of the young generation.

## References

[cit0001] Aerts D., Apostel L., Moor B. D., Hellemans S., Maex E., Va Belle H, Veken J. V. (2007). World views: From fragmentation to integration.

[cit0002] Albert D., Steinberg L., Bardo M. T., Fishbein D. H., Milich R. (2011). Peer influences on adolescent risk behavior.

[cit0003] Albrecht S. L., Chadwick B. A., Alcorn D. S. (1977). Religiosity and deviance: Application of an attitude–behaviour contingent consistency model. Journal for the Scientific Study of Religion.

[cit0004] Andersson J. (2011). Vauxhall's post-industrial pleasure gardens: ‘Death wish’ and hedonism in 21st-century London. Urban Studies.

[cit0005] Armsden G. C., Greenberg M. T. (1987). The inventory of parent and peer attachment: Individual differences and their relationships to psychological well-being in adolescence. Journal of Youth and Adolescence.

[cit0006] Arnett J. J. (2000). Emerging adulthood: A theory of development from the late teens through the twenties. American Psychologist.

[cit0007] Azimi H., Turiman S., Ezhar T. (2000). Gaya hidup dan gaya fikir belia dalam pembangunan negara, dalam Belia dalam menghadapi alaf baru. [Youth lifestyle and thinking pattern in national development faces the new millennium].

[cit0009] Bosnia H. A., Kunnen E. S. (2001). Determinants and mechanisms in ego-identity development: A review and synthesis. Developmental Review.

[cit0008] Bowlby J. (1969). Attachment and loss, vol. 1: Attachment.

[cit0010] Brechwald W. A., Prinstein M. J. (2011). Beyond homophily: A decade of advances in understanding peer influence processes. Journal of Research on Adolescence.

[cit0011] Bronfenbrenner U. (1979). The ecology of human development: Experiments by nature and design.

[cit0012] Bronfenbrenner U., Vasta R. (1989). Ecological systems theory.

[cit0013] Browne M. W., Cudeck R., Bollen K. A., Long J. Scott (1993). Alternative ways of assessing model fit.

[cit0014] Byrne B. M. (2010). Structural equation modeling with AMOS: Basic concepts, applications, and programming.

[cit0015] Chaudhuri A., Aboulnasr K., Ligas M. (2010). Emotional responses on initial exposure to a hedonic or utilitarian description of a radical innovation. Journal of Marketing Theory and Practice.

[cit0016] Cheng T. L. (2013). Attitudes towards work, life, career and the world view: Three generational perspectives across Malaysia. Journal of Business Management and Administration.

[cit0017] Cotton S., Zebracki K., Rosenthal S. L., Tsevat J., Drotar D. (2006). Religion/spirituality and adolescent health outcomes: A review. Journal of Adolescent Health.

[cit0018] Crisp R. (2006). Reasons and the good.

[cit0019] Cullman E. P. (2006). Attachment to parent and peers as a moderator of the relation between parent/peer religious coping and adolescent religious coping.

[cit0020] Deckard S., DeWitt D. (2003). Worldview studies book one: Developing a creator-centered worldview.

[cit0021] Department of Statistic Malaysia (2010). http://www.statistics.gov.my/portal/images/stories/files/latestreleases/population/findings_2010-2040.pdf.

[cit0022] Desmond S. A., Morgan K. H., Kikuchi G. (2010). Religious development: How (and why) does religiosity change from adolescence to young adulthood?. Sociological Perspectives.

[cit0023] Durlak J. A., Weissberg R. P., Pachan M. (2010). A meta-analysis of after-school programs that seek to promote personal and social skills in children and adolescents. American Journal of Community Psychology.

[cit0024] Dykas M. J., Ziv Y., Cassidy J. (2008). Attachment and peer relations in adolescence. Attachment & Human Development.

[cit0025] Feldman F. (2008). Whole life satisfaction concepts of happiness. Theoria.

[cit0026] French D. C., Purwono U., Rodkin P. C. (2012). Religiosity of adolescents and their friends and network associates: Homophily and associations with antisocial behaviour. Journal of Research on Adolescence.

[cit0027] Glaser B., Shelton H. K., Bree M. (2010). The moderating role of close friends in the relationship between conduct problems and adolescent substance use. Journal of Adolescent Health.

[cit0028] Hair J. F. J., Black W. C., Babin B. J., Anderson R. E. (2010). Multivariate data analysis.

[cit0060] Hamzah S. R., Krauss S., Suandi T., Hamzah A., Tamam E. (2013). The moderating effect of parent and peer influences on hedonistic behavior among undergraduate student in Malaysia. Asian Social Science.

[cit0029] Hartup W. (1996). The company they keep: Friendships and their developmental significance. Child Development.

[cit0030] Hu L., Bentler P. M. (1999). Cutoff criteria for fit indexes in covariance structure analysis: Conventional criteria versus new alternatives. Structural Equation Modeling.

[cit0031] Huang Y., Wang L., Shi J. (2012). How attachment affects the strength of peer influence on adolescent consumer behaviour. Psychology & Marketing.

[cit0032] Itafarida (2008). The impact of sociocultural changes on the youth?s achievement orientation in Fitzgerald?s the beautiful and damned. k@ta.

[cit0033] Setiadi B. N., Supratiknya A., Lonner W. J., Poortinga Y. H., Jaafar (2004). The impact of cultural norms and values on the moral judgment of Malay and American adolescents: A brief report. http://www.iaccp.org.

[cit0035] Jantzen C., Fitchett J., Østergaard P., Vetner M. (2012). Just for fun? The emotional regime of experiential consumption. Marketing Theory.

[cit0034] Jas Laile Suzana J. (2008). Introduction to children and adolescents psychology.

[cit0036] Johnson B. R., Jang S. J., Larson D. B., De Li S. (2001). Does adolescent religious commitment matter? A reexamination of the effects of religiosity in delinquency. The Journal of Research in Crime and Delinquency.

[cit0037] Kandel D. B. (1978). Homophily, selection and socialization in adolescent friendships. American Journal of Sociology.

[cit0038] Kearney M. (1984). World view.

[cit0039] Keats D. M. (2000). Cross-cultural studies in child development in Asian contexts. Cross-Cultural Research.

[cit0040] King P. E., Furrow J. L. (2008). Religion as a resource for positive youth development: Religion, social capital, and moral outcomes. Psychology of Religion and Spirituality.

[cit0041] Krauss S. E., Azimi H., Fazilah I. (2007). Adaptation of a Muslim religiosity scale for use with four different faith communities in Malaysia. Review of Religious Research.

[cit0043] Krauss S. E., Hamzah A. H., Suandi T., Noah S. M., Juhari R., Manap J. H., Mahmood A. (2006). Exploring regional differences in religiosity among Muslim youth in Malaysia. Review of Religious Research.

[cit0042] Krauss S. E., Ismail I. A., Suandi T., Hamzah A., Hamzah S. R., Dahalan D., Idris F. (2013). Parenting and community engagement factors as predictors of religiosity among Muslim adolescents from Malaysia. International Journal for the Psychology of Religion.

[cit0044] Kroger J. (2000). Identity development: Adolescence through adulthood.

[cit0046] Liu R. X. (2011). Strain as a moderator of the relationship between parental attachment and delinquent participation: A China study. International Criminal Justice Review.

[cit0045] Lynam D. R., Loeber R., Stouthamer-Loeber M. (2008). The stability of psychopathy from adolescence into adulthood: The search for moderators. Criminal Justice and Behaviour.

[cit0047] Malaysian Youth Index (2011). Malaysian Institute for Research in Youth Development.

[cit0048] Maria C., Elias H., Mahyudin R., Uli J. (2009). Adjustment amongst first year students in a Malaysian university. European Journal of Social Sciences.

[cit0049] Mcelhaney K. B., Immele A., Smith F. D., Allen J. P. (2006). Attachment organization as a moderator of the link between friendship quality and adolescent delinquency. Attachment & Human Development.

[cit0050] Ministry of Higher Education (2010). Statistics of Higher Education of Malaysia 2010.

[cit0051] Minkov M. (2007). What makes us different and similar: A new interpretation of the World Values Survey and other cross-cultural data.

[cit0052] Pargament K. I., Park C. L. (1995). Merely a defense? The variety of religious means and ends. Journal of Social Issues.

[cit0053] Piaget J. (1965). The moral judgment of the child.

[cit0054] Regnerus M. D., Smith C., Smith B. (2004). Social context in the development of adolescent religiosity. Applied Developmental Science.

[cit0055] Ross R. (1962). Symbols and civilization: Science, morals, religion, art.

[cit0056] Rubin K. H., Bukowski W. M., Parker J. G., Eisenberg N., Damon W., Lerner R. M. (2006). Peer interactions, relationships, and groups.

[cit0057] Salzinger S., Feldman R. S., Rosario M., Ng-Mak D. S. (2010). Role of parent and peer relationships and individual characteristics in middle school children's behavioural outcomes in the face of community violence. Journal of Research on Adolescence.

[cit0058] Scholte R. H. J., Van Aken M. A. G., Jackson S., Goossens L. (2006). Peer relations in adolescence.

[cit0059] Sinha J. W., Cnaan R. A., Gelles R. W. (2007). Adolescent risk behaviours and religion: Findings from a national study. Journal of Adolescence.

[cit0061] Sobel D. (2002). Varieties of hedonism. Journal of Social Philosophy.

[cit0062] Stolz H. E., Olsen J. O., Henke T. M., Barber B. K. (2013). Adolescent religiosity and psychosocial functioning: Investigating the roles of religious tradition, national-ethnic group, and gender. Child Development Research.

[cit0063] Thorpe H. (2012). Sex, drugs and snowboarding: Legitimate definitions of taste and lifestyle in a physical youth culture. Leisure Studies.

[cit0064] Tomé G., Matos M. G., Simões C., Camacho I., Diniz J. A. (2012). How can peer group influence the behaviour of adolescents: Explanatory model. Global Journal of Health Science.

[cit0065] Urberg K. A., Luo Q., Pilgrim C., Degirmencioglu S. M. (2003). A two-stage model of peer influence in adolescent substance use: Individual and relationship-specific differences in susceptibility to influence. Addictive Behaviours.

[cit0066] Veenhoven R. (2003). Hedonism and happiness. Journal of Happiness Studies.

[cit0067] Walden L. M., Beran T. N. (2010). Attachment quality and bullying behaviour in school-aged youth. Canadian Journal of School Psychology.

[cit0068] Waldrip A. M., Malcolm K. T., Jensen-Campbell L. A. (2008). With a little help from your friends: The importance of high-quality friendships on early adolescent adjustment. Social Development.

[cit0070] Wallace J. M., Forman T. A. (1998). Religion's role in promoting health and reducing risk among American youth. Health Education Behaviour.

[cit0069] Wallace J. M., Williams D. R., Schulenberg J., Maggs J., Hurrelmann K. (1997). Religion and adolescent health compromising behaviour.

[cit0071] Weijers D. (2011). Hedonisme, Internet encyclopedia of philosophy. http://www.iep.utm.edu/hedonisme/.

[cit0072] Zahrt D. M., Lange M. D. (2011). Aggressive behaviour in children and adolescents. Pediatrics in Review.

[cit0073] Zimbardo P. G., Boyd J. N. (1999). Putting time in perspective: A valid, reliable, individual-differences metric. Journal of Personality and Social Psychology.

